# Specific Responses of *Salmonella enterica* to Tomato Varieties and Fruit Ripeness Identified by *In Vivo* Expression Technology

**DOI:** 10.1371/journal.pone.0012406

**Published:** 2010-08-31

**Authors:** Jason T. Noel, Nabil Arrach, Ali Alagely, Michael McClelland, Max Teplitski

**Affiliations:** 1 Soil and Water Science Department, Genetics Institute, University of Florida, Gainesville, Florida, United States of America; 2 Vaccine Research Institute of San Diego, La Jolla, California, United States of America; 3 Sanford-Burnham Medical Research Institute, La Jolla, California, United States of America; 4 Department of Pathology and Laboratory Medicine, University of California Irvine, Irvine, California, United States of America; University of Hyderabad, India

## Abstract

**Background:**

Recent outbreaks of vegetable-associated gastroenteritis suggest that enteric pathogens colonize, multiply and persist in plants for extended periods of time, eventually infecting people. Genetic and physiological pathways, by which enterics colonize plants, are still poorly understood.

**Methodology/Principal Findings:**

To better understand interactions between *Salmonella enterica* sv. Typhimurium and tomatoes, a *gfp*-tagged *Salmonella* promoter library was screened inside red ripe fruits. Fifty-one unique constructs that were potentially differentially regulated in tomato relative to *in vitro* growth were identified. The expression of a subset of these promoters was tested *in planta* using recombinase-based *in vivo* expression technology (RIVET) and fitness of the corresponding mutants was tested. Gene expression in *Salmonella* was affected by fruit maturity and tomato cultivar. A putative *fadH* promoter was upregulated most strongly in immature tomatoes. Expression of the *fadH* construct depended on the presence of linoleic acid, which is consistent with the reduced accumulation of this compound in mature tomato fruits. The *cysB* construct was activated in the fruit of cv. Hawaii 7997 (resistant to a race of *Ralstonia solanacearum*) more strongly than in the universally susceptible tomato cv. Bonny Best. Known *Salmonella* motility and animal virulence genes (*hilA, flhDC, fliF* and those encoded on the pSLT virulence plasmid) did not contribute significantly to fitness of the bacteria inside tomatoes, even though deletions of *sirA* and *motA* modestly increased fitness of *Salmonella* inside tomatoes.

**Conclusions/Significance:**

This study reveals the genetic basis of the interactions of *Salmonella* with plant hosts. *Salmonella* relies on a distinct set of metabolic and regulatory genes, which are differentially regulated *in planta* in response to host genotype and fruit maturity. This enteric pathogen colonizes tissues of tomatoes differently than plant pathogens, and relies little on its animal virulence genes for persistence within the fruit.

## Introduction

The increase in produce-associated gastroenteritis outbreaks indicates that non-typhoidal serovars of *Salmonella enterica* and enterovirulent *E. coli* can contaminate fruit, vegetables and sprouts [1]–[3]. *S. enterica* (including sv. Typhimurium 14028) and enterovirulent *E. coli* were found to colonize internal tissues of tomato, lettuce, alfalfa, cilantro, where they reach population levels as high as 10^5^–10^7^ cfu/g of plant tissue under field and/or laboratory conditions [4]–[7]. These populations are approximately two orders of magnitude lower than those reached by dedicated phytopathogens like *Pectobacterium carotorovum* [7]. These observations indicate that under most biologically relevant conditions, *Salmonella* can efficiently utilize nutrients found within plant tissues. However, behaviors and nutrition of enterics inside plants are not well understood.

Unlike closely related plant-associated members of the *Enterobacteriaceae*, *Salmonella enterica* and *E. coli* do not produce enzymes capable of degrading plant cell wall polymers and their genomes do not encode homologs of known pectinases or hemicellulases [8], [9]. Therefore, to proliferate inside plant tissues, these enteric pathogens may rely on their ability to acquire simpler carbon sources. This hypothesis was recently supported by a transcriptomic analysis of the *E. coli* O157:H7 growth on lettuce leaf lysates [10]. Tomato fruits contain simple sugars, sugar alcohols, organic and fatty acids, and amino acids, which could be utilized by *Salmonella*. The amounts of these metabolites differ among cultivars and also depend on the maturity stage of fruit [11], [12].

To formulate a hypothesis about behavior of *S. enterica* within plant tissues, we analyzed reports of gene regulation in plant pathogens during their interactions with plant hosts. *In planta* gene expression has been documented for *Xanthomonas campestris* pv vesicatoria during formation of a leaf spot on tomato [13], *Ralstonia solanacearum* during bacterial wilt of tomato [14], the fire blight pathogen *Erwinia amylovora* during rot of immature pear fruits [15], *Pseudomonas syringae* pv syringae during colonization of bean phyllosphere [16] and *P. syringae* pv tomato during growth inside tissues of *Arabidopsis thaliana* [17]. Even though screen conditions differed, all of these phytopathogens were found to express a common set of ∼5–15% of their genes during interactions with plant hosts. These commonly expressed genes included those for type III secretion systems and effectors translocated by them, as well as genes involved in the degradation of plant polymers. A significant number of differentially regulated genes were involved in stress resistance and responses to antibiotics, uptake of iron, amino and carboxylic acids and synthesis of amino acids and proteins [13], [14], [17]. Therefore, if *Salmonella* behaves similarly to plant-associated bacteria during colonization of plant tissues, it is reasonable to expect differential regulation of genes corresponding to similar functions.

A few of the genes and mechanisms that enteric bacteria use to colonize external surfaces of host plants have been identified. Bacterial polymers (cellulose, poly-β-1,6-*N*-acetyl-D-glucosamine, colanic acid and O-antigen capsule) and aggregative fimbriae are involved in the attachment of *E. coli* and/or *Salmonella* to plant surfaces [18]–[23]. In their reliance on self-produced cellulose for attachment to plant surfaces, enteric pathogens are similar to the plant symbiotic and pathogenic bacteria but differ from the interactions of *Salmonella* with animal tissues, where cellulose production is dispensable [24].

Understanding the behavior and nutrition of non-typhoidal *Salmonella enterica* inside plants may reveal ecological strategies this enteric pathogen uses to persist outside its animal hosts. Defining differences in the *Salmonella* survival in different tomato cultivars or maturity stages may offer opportunities to modify pre- or post-harvest practices for promoting safer produce.

## Results

### *Salmonella enterica* sv. Typhimurium 14028 as a model

In a preliminary study, we tested whether *S. enterica* sv. Typhimurium ATCC 14028 would serve as a useful model for this study. Several serovars of non-typhoidal *S. enterica* have been linked to multi-state outbreaks of produce-borne salmonellosis, however only one isolate of *S. enterica* sv. Typhimurium was linked to a “tomato” outbreak [25]. *S. enterica* sv Typhimurium was the prevalent (91%) serovar isolated from fruit rinses, irrigation and drinking water in a comprehensive survey of multiple farms in Mexico [26]. Collectively, these reports suggest that studies using this common serovar may have ecological significance. Even though genome sequences are becoming available for a number of the outbreak strains, the genetic and genomic tools available for *S. enterica* sv Typhimurium ATCC 14028 are unmatched [27].

To test whether ATCC 14028 colonizes tomato fruit tissues as well as the strains isolated from the salmonellosis outbreaks linked to the tomato consumption, proliferation of this strain inside red ripe tomatoes (cv. Campari) was compared with that of the six-strain cocktail containing of *S. enterica* sv. Newport, Braenderup, Javiana and Montevideo, which were isolated from tomato-related salmonellosis outbreaks or from tomato fields on the Eastern Shore of Virginia (Table S1).

**Figure 1:**
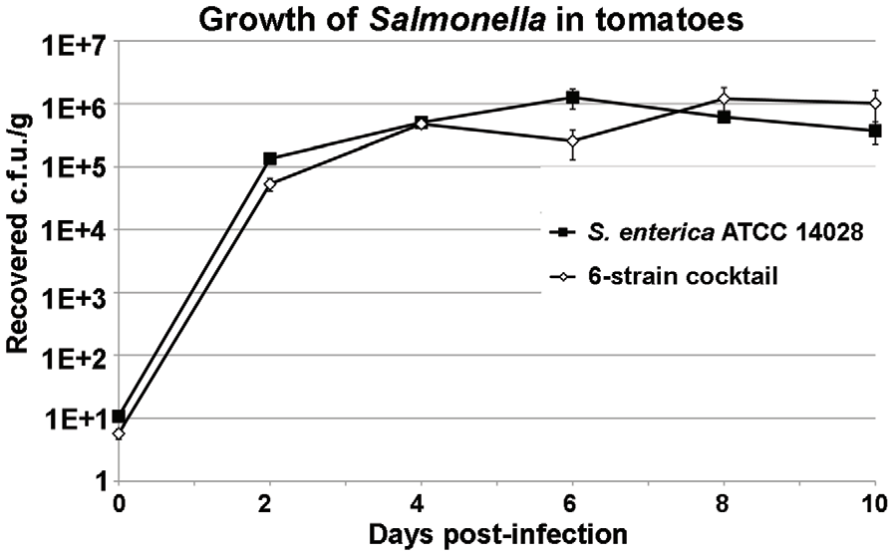
Growth of *Salmonella enterica* strains inside red ripe tomatoes. *S. enterica* sv. Typhimurium ATCC 14028 or a six-strain cocktail containing *S. enterica* sv. Newport C6.3, sv. Braenderup 04E01347, Braenderup 04E01556, Braenderup 04E00783, Montevideo LJH519 and sv. Javiana ATCC BAA-1593 were seeded onto shallow wounds in red ripe tomatoes of cv. Campari. Tomatoes were incubated at 22°C, 40–60% relative humidity. At indicated time points, three tomatoes per treament were harvested, blended for 1 min at 260 r.p.m. in Stomacher 400 Circulator (Seward, West Sussex, U.K.), and aliquots were plated on XLD medium. Averages of the three samples are shown, error bars are standard errors.

As shown in Fig. 1, *S. enterica* sv. Typhimurium ATCC 14028, the model for this research, reached approximately 10^6^ c.f.u./g of tomato tissue, and grew inside tomatoes to the same final densities as the outbreak strains (Fig. 1). These observations are consistent with the report that another isolate of *S.* Typhimurium colonized tomato fruits similarly to the strains, which have been linked to the gastroenteritis outbreaks [28]. While it is likely that there exist strain-specific differences in the regulation of some *Salmonella* genes during colonization of tomatoes, the ability of ATCC 14028 to proliferate inside tomatoes as efficiently as the outbreak strains indicates that the data obtained using this model will be useful in understanding the role of non-host environments in the ecology of *Salmonella*.

### Survey of *Salmonella* genes induced in tomato fruit

**Figure 2:**
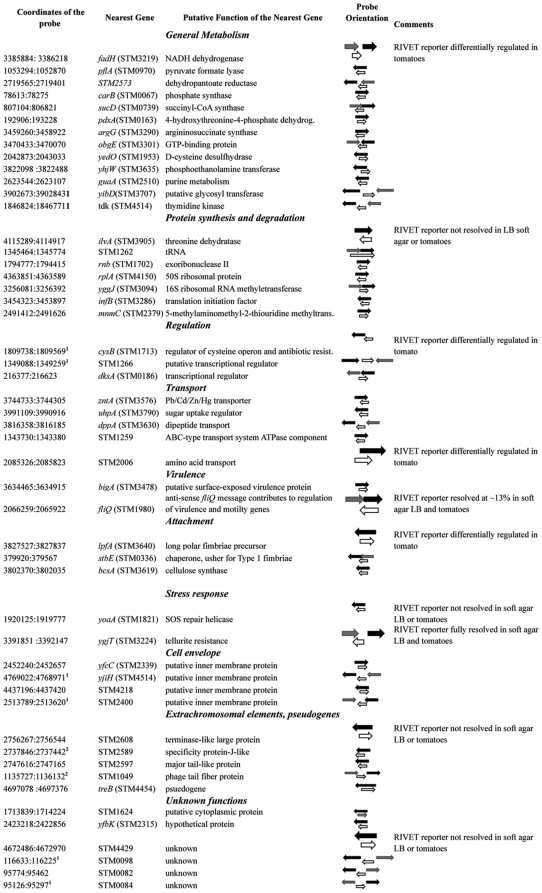
Probes differentially regulated inside red ripe tomato. Fragments of *Salmonella* genomic DNA which effected differential accumulation of the GFP reporter protein were identified by sequencing, their coordinates are listed in the far left column. Coordinates are based on the *S. enterica* sv Typhimurium genome sequence, GenBank accession # NC_003197. The position and orientation of the probe are indicated as an unfilled arrow. The nearest annotated ORFs are shown as grey or black arrows. Sequences indicated with ‘1’ occur multiple times within the *Salmonella* genome; sequences indicated with ‘2’ occur twice in the *Salmonella* genome.

To identify promoters that are strongly regulated in *Salmonella* during colonization of tomato fruits, two promoter-*gfp* libraries were screened and tomato-specific constructs were isolated. Two hundred eighty-eight clones containing differentially regulated promoters were identified and sequenced. Fifty-one unique fragments differentially regulated during growth inside tomato fruit were identified (Fig. 2). Six concatemer constructs were identified and discarded (data not shown).

To validate results of the FACS sort, promoter-*tnpR* RIVET reporters in several representative genes were constructed (Fig. 2). TnpR-based RIVET reporters have been shown to be sensitive and reliable for quantifying *Salmonella* gene expression in animal and plant hosts, including tomato [7], [29]. Regulation of the promoters of interest in tomato fruits of cultivars harvested at two different maturity stages was tested. Below, we will consider only those select reporters that were validated by RIVET assays.

### The promoter adjacent to *fadH* is differentially expressed in green vs red fruits

**Figure 3:**
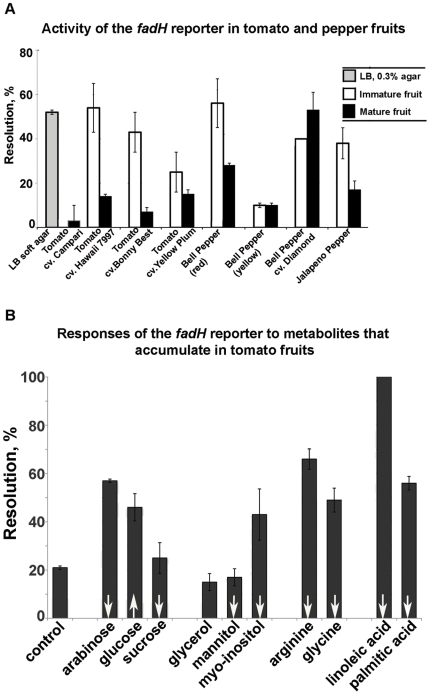
*fadH* expression depends on fruit ripeness and a specific metabolite. (**A**) To test regulation of *fadH* inside fruits of tomatoes and peppers, *fadH+ fadH::tnpR res1-tet-res1* reporter was seeded onto shallow wounds in mature and immature fruits of tomatoes and peppers. Activity of the reporter was scored as the loss of the tetracycline marker in the recovered bacteria (‘resolution’). Averages of at least two biological and three technical replications are shown, error bars are standard errors. Fruits of each cultivar at different maturity stages were collected from the same plant. In LB broth, resolution of the *fadH* RIVET reporter was 0%. (**B**) To test which metabolite from red or green tomatoes affects the *fadH* RIVET reporter, resolution of the reporter was measured in soft M9 agar (0.3%) supplemented with the metabolites present in tomato fruits. An upward white arrow at the bottom of each bar indicates that the metabolite increases as tomato matures, a downward arrow indicates that the metabolite decreases with the maturity of the fruit [11]. Glycerol is present at the same amounts in red and green fruit [11], and therefore is not marked with an arrow. The control sample contained no carbon sources. The experiment was repeated twice, with two technical replications. Data from the second experiment are shown, error bars are standard errors.

The resolution of the RIVET reporter (*S. enterica* sv. Typhimurium JTN24) in the promoter adjacent to *fadH* was strongly reduced in red ripe tomatoes compared to the soft agar LB (Fig. 3A). Consistent with the FACS experiments, there was no resolution of the construct in LB broth (data not shown). The *fadH* gene encodes 2,4-dienoyl-CoA reductase, an iron-sulfur flavoenzyme required for the metabolism of unsaturated fatty acids with double bonds at even carbon positions [30]. In fruits that are green when immature and turn red when mature (tomato cvs. Hawaii 7997, Bonny Best; jalapeño and red bell pepper), the resolution of the *fadH* RIVET reporter was significantly higher in green fruit compared to red ripe fruit. Interestingly, in bell pepper cv. Diamond (ivory when immature, red when mature), resolution of the reporter was strong in mature and immature fruit (Fig. 3A). In fruit of tomato and bell pepper that are green when immature and yellow when mature, expression was low and not statistically different between fruits of different cultivars (Fig. 3A). The reporter was not resolved on surfaces of leaves of tomato cvs Bonny Best or Hawaii 7997 (data not shown). These observations suggested that the reporter responds either positively or negatively to the presence of chemical cues in fruit at different maturity stages.

To test the hypothesis that the expression of the promoter upstream of *fadH* depended on the presence of carotenoid pigments, which accumulate as the fruits ripen and turn red, hydrophobic pigments were extracted with chloroform. Resolution of the reporter was tested in soft LB agar (0.3%) supplemented with the tomato extract. No difference in the activity of the reporter was observed in response to the hydrophobic extract of red ripe tomatoes: 80±7% (± standard error) in soft LB agar with tomato extract vs 74±6% in soft LB agar. This indicates that it was not non-polar compounds found in ripe tomatoes, which down-regulated the reporter. Rather, it is likely to be a substance present in green tomatoes that activated the reporter.

### Linoleic acid upregulates *Salmonella* promoter adjacent to *fadH*

In red ripe fruit, sucrose content decreases while glucose increases. Palmitic and linoleic acids, myo-innositol, mannitol, glycine, arginine and arabinose are found in higher amounts in immature fruit (compared to a red ripe fruit) [11]. To test whether any of these compounds affect expression of the *fadH+ fadH::tnpR* RIVET reporter JTN24, bioassays were carried out with each of these compounds spotted on glass fiber disks and placed on the surface of soft agar based on M9 minimal medium seeded with the reporter. As an additional control, one of the assays contained glycerol (its concentration in the tomato fruit does not change as the fruit matures [11]). As shown in Fig. 3B, the basal level of the reporter resolution in M9 soft agar was approximately 20%. The addition of sucrose (which is not metabolized by *S. enterica* [31]), mannitol or glycerol did not affect regulation of the reporter (Fig. 3B). Partial induction was observed in the presence of other tomato metabolites (arabinose, arginine, glucose, glycine, myo-innositol and palmitate) known to differentially accumulate in green (and not ripe) tomatoes. The addition of linoleic acid fully induced the reporter (Fig. 3B) indicating that this is likely the cue that triggers the expression of the *fadH* RIVET reporter in green, and not red ripe tomatoes.

### Tomato genotype contributes to the regulation of *Salmonella cysB*

**Figure 4:**
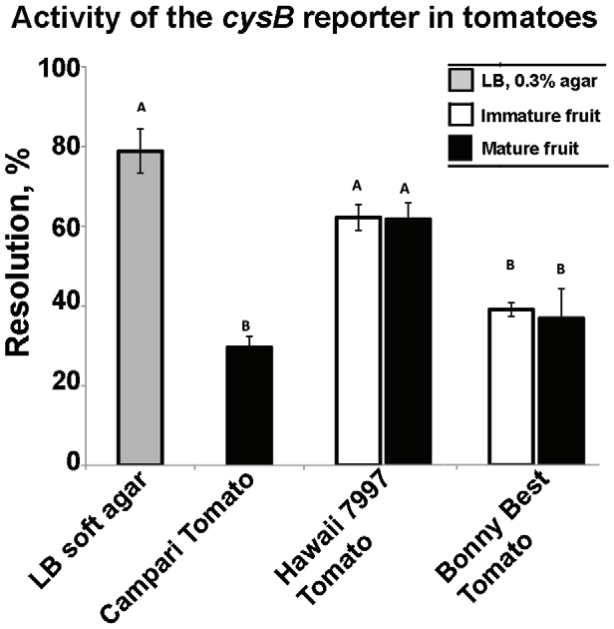
Tomato cultivar-dependent regulation of *Salmonella cysB*. Activity of the chromosomal *cysB+ cysB::tnpR res1-tet-res1* reporter was scored as the loss (‘resolution’) of tetracycline marker flanked by *res* sites in reporter cells recovered from red and green tomatoes of three cultivars and soft LB agar. Resolution of the reporter in LB broth was 2±2%. Averages of at least two biological and three technical replications are shown, error bars are standard errors. Data points that are significantly different are indicated by different upper case letters; statistical significance was established by 1-way ANOVA and Tukey's Honestly Significant Difference (HSD) for post hoc comparison of means. All statistical tests were performed using JMP 5.1.2 (SAS Institute, Inc).

A fragment spanning the *cysB* promoter [32] was identified as differentially regulated inside tomatoes (Fig.2). Consistent with this, resolution of the *cysB* RIVET reporter (*S. enterica* sv. Typhimurium JTN71) in LB broth was 2±2% and was increased in tomato fruits (Fig. 4). No resolution of the reporter was observed in the phyllosphere of cvs. Bonny Best or Hawaii 7997 (data not shown). The *cysB* gene encodes a regulator of an operon involved in cysteine acquisition [32], [33]. Cysteine accumulates in red ripe tomatoes [11], therefore we tested whether the expression of the *cysB* RIVET reporter would depend on the maturity of the fruit. As shown in Fig. 4, regulation of the *cysB* reporter was not affected by maturity of the fruit; rather it depended on the cultivar in which the reporter was tested. The expression of *cysB* was highest in the fruit of cv. Hawaii 7997 which is resistant to a race of a vascular pathogen *R. solanacearum* [34]. Expression of the *cysB* reporter was lower in the fruits of cvs. Campari and Bonny Best which are both susceptible to a number of bacterial and fungal pathogens. The correlation between the expression of *cysB* and tomato disease-resistant genotype is intriguing because one of the *cysB*-dependent phenotypes in *S. enterica* is resistance to antibiotics (although the mechanism by which L-cysteine and *cysB* effect this behavior is not entirely clear) [33]. Interestingly, genes involved in cysteine synthesis were differentially regulated in *P. syringae* on bean leaf surfaces [16] and in *E. coli* O157:H7 in response to lettuce leaf lysates [10]. These observations suggest a conserved function for the cysteine synthesis genes in the interactions of enterics and plant pathogens with their plant hosts.

### Regulation of STM2006

**Figure 5:**
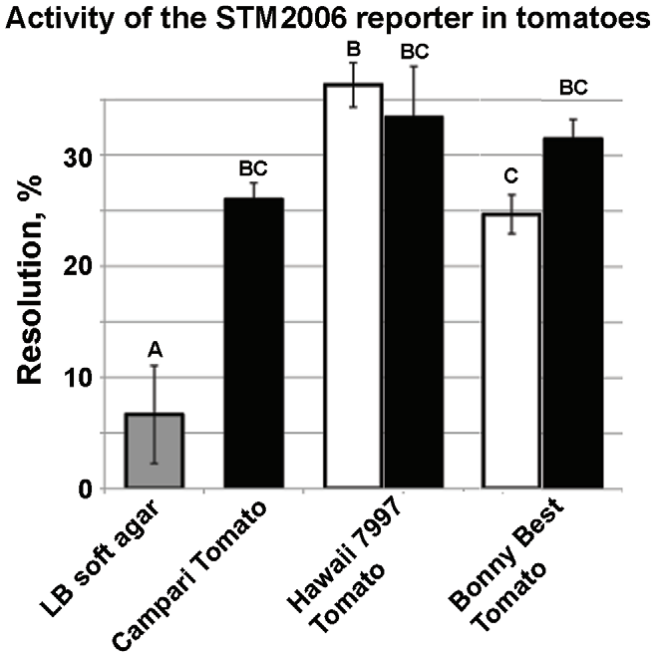
Activity of the STM2006 RIVET reporter in tomatoes. *cis* merodiploid chromosomal RIVET reporter in STM2006 was upregulated in tomatoes, compared to soft LB agar. Averages of the two biological and three technical replications are shown; tomatoes of each cultivar at different maturity stages were harvested from the same plant at the same time. Data points that are significantly different are indicated by different upper case letters; statistical significance was established by 1-way ANOVA and Tukey's Honestly Significant Difference (HSD) for post hoc comparison of means. All statistical tests were performed using JMP 5.1.2 (SAS Institute, Inc).

STM2006 is predicted to encode a DNA-binding prophage protein, which is found in the genomes of some salmonellae, with a close homologue in *Pectobacterium carotovorum*, a plant-associated member of *Enterobacteriacea*. Expression of the STM2006 RIVET reporter (*S. enterica* sv. Typhimurium JTN69) was 3-to-4 fold higher inside red or green tomatoes of three cultivars compared to the LB control (Fig. 5). No resolution of the reporter was observed on tomato leaf surfaces (data not shown). Because orthologs of STM2006 are annotated as amino acid transporters in some *Salmonella* published genomes, we tested whether supplementation of M9-glucose medium with any of the 20 amino acids or γ-aminobutyric acid would mimic the increased expression observed in tomatoes. None of tested amino acids (at 0.5-1 mM) affected expression of the reporter in STM2006 to an appreciable degree (data not shown). Therefore, while STM2006 is clearly upregulated inside tomatoes, the mechanism of this differential regulation is not yet clear.

### Cryptic genes

The analysis of the genomic context revealed that only eleven of the clones identified by FACS included known promoters (Fig. 2). The other constructs represent intergenic regions or “cryptic” genes, in which a differentially regulated fragment overlaps the ORF or is in the opposite orientation (Fig. 2). This was not unexpected. Such cryptic constructs are commonly identified using *in vivo* expression analyses and represent a sizable proportion of the *in vivo* expressed genes [13], [14], [16], [35]. We do not yet know whether these sequences are simply “noise” inherent to high-throughput assays or whether these are indications of niche-specific gene regulation through alternate mechanisms.

To test whether fragments found within ORFs, including those in “backwards” orientations, acted as inducible promoters, chromosomal RIVET reporters were constructed in *fliQ*, *ilvA*, STM4429, STM2608 and *lpfA* (Fig. 2). Regulation of these single copy reporters was tested in soft LB agar (0.3%) and in tomatoes. The single copy chromosomal reporters in *ilvA,* STM4429 and STM2608 did not resolve inside red ripe tomato or in soft LB agar (Fig. 2). The RIVET reporter corresponding to the antisense *fliQ* transcript was resolved at ∼13% in soft LB agar and in tomatoes (Fig. 2). This is consistent with the report by Wang and Harshey (2009), which identified low level antisense transcription of the *fliPQR* cistron [36].

**Figure 6:**
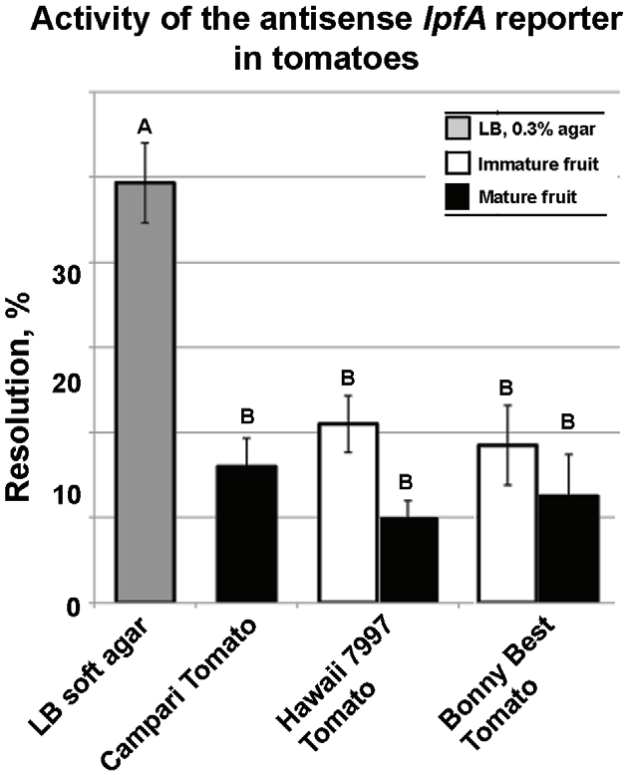
Regulation of the RIVET reporter in the internal anti-sense fragment of *lpfA* in tomatoes. A chromosomal RIVET reporter constructed within *lpfA* and in the antisense orientation was strongly expressed in soft LB agar and down-regulated in tomatoes, regardless of the maturity state or the cultivar. In LB broth, the reporter was resolved at 5±3%. Averages of the two biological and three technical replications are shown; tomatoes of each cultivar at different maturity stages were harvested from the same plant at the same time. Data points that are significantly different are indicated by different upper case letters; statistical significance was established by 1-way ANOVA and Tukey's Honestly Significant Difference (HSD) for post hoc comparison of means.

The “backwards” RIVET reporter in *lpfA* was expressed at 5±3% in LB broth and at 49%±5% in soft LB agar (0.3%) (data not shown and Fig. 6). Expression of this reporter was reduced inside tomatoes, the regulation of the cryptic promoter within *lpfA* was not affected by the cultivar or maturity of the tomato. The mechanism of this regulation inside tomatoes is not yet clear, nor is it clear how important the anti-sense regulation is to the interactions of *Salmonella* with its hosts.

### Fitness of the mutants in the tomato-dependent genes

We constructed individual deletion mutants in *fadH, cysB* and in the first genes in the following putative cistrons: STM2006*-*STM2008; and *lpfABCDE-yhjW-proK*. We tested competitive fitness of these and *bcs* [37] mutants within red ripe tomatoes. As indicated in Table 1, with the exception of *cysB,* none of the single mutants was significantly less fit than the wild type in the red ripe fruit of tomato cv. Campari. Competitive fitness of the Δ*cysB* mutant in fruits of cv. Hawaii 7997 and Bonny Best was not statistically different from the control (data not shown), and the competitive fitness of the Δ*cysB* mutant was only modestly reduced in red ripe tomatoes of cv. Campari. A mutant, in which *bcsA, cysB, fadH, lpfA* and STM2006 were all deleted, was modestly, but statistically significantly less fit than the wild type in tomatoes (Table 1), but not in LB shake cultures (data not shown). It is possible that the relevant genes being regulated by the promoters were downstream of these proximal genes. Alternatively, or possibly concurrently, persistence of *Salmonella* inside tomatoes is a complex phenotype, controlled by multiple nutritional and regulatory inputs that are redundant, making the expression of individual genes less important.

**Table 1:** Fitness of *Salmonella enterica* mutants in red ripe tomatoes, cv Campari.

Genotypes	Mean of the log competitive index ± standard error
*ΔfadH15::kan*	0.170±0.330
*Δbcs::kan*	−0.156±0.145
*Δ* ***cysB22::kan*** 1	**−0.276±0.086**
*ΔlpfA30::kan*	−0.010±0.280
*Δ*STM2006::*kan*	0.300±0.310
*Δ* ***bcs*** * Δ* ***lpfA30*** * Δ* ***fadH15*** *Δ* ***STM2006*** * Δ* ***cysB22::kan***	**−0.510±0.010**

1 –boldface shading indicates that the strain is less competitive than the wild type, based on a two-tailed t-test (p<0.05) of log competitive indices as described in Materials and Methods.

### Role of known virulence, motility and attachment genes in persistence within fruit

In *E. coli* O157:H7, known virulence genes were upregulated within the first 15–30 minutes of exposure to lettuce leaf exudates [10]. *Salmonella* virulence gene were also involved in the colonization of alfalfa seedlings [19], [38], however promoters of the *Salmonella* virulence genes were largely absent from the list in Fig. 2. This could be explained by the fact that animal virulence genes were not differentially regulated inside tomato fruit. Alternatively, the corresponding constructs may have been eliminated during the pre-sorting step (after growth in LB broth and prior to inoculation into tomatoes) because many virulence genes are expressed in LB medium and the corresponding promoter probes would have been eliminated during the pre-sorting step.

The majority of *Salmonella* strains recovered from fruit- or vegetable-related outbreaks produced curli [23]. Genes encoding aggregative fimbriae (curli) were shown to be involved in binding of *Salmonella* and some *E. coli* strains to alfalfa seedlings and lettuce leaves [18]–[22]. However, none of the genes in the *agf* operon were identified in our screen. To test whether aggregative fimbriae are involved in the persistence of *Salmonella* within tomatoes, we constructed a mutant with deletions in the *agfC* and *agfB* genes, and tested the expression of the *agf* RIVET reporter (*S. enterica* sv. Typhimurium JTN204) inside tomatoes. The resolution of the JTN204 RIVET reporter in LB shake cultures was 46±9%, while inside red ripe Campari tomatoes the resolution was significantly lower (5±2%). This lack of up-regulation of the reporter inside the tomato is consistent with the results of the FACS sort, which did not identify *agf* promoter as differentially regulated inside tomato. Competitive fitness of the mutant lacking *agfB* and *agfC* was indistinguishable from that of the wild type (Table 2), consistent with the lack of expression of the gene inside tomatoes. Conversely, a RIVET reporter in the *yihT* gene (*S. enterica* sv. Typhimurium JTN203) encoding an aldolase involved in capsule synthesis was expressed in red ripe Campari tomatoes (27±4%), but not in LB. However, deletion of *yihT* or of the entire *yihT-ompL* region did not reduce competitive fitness of the strain in red ripe tomatoes (Table 2). These results indicate that even though aggregative fimbriae and capsule gene were involved in attachment to plant surfaces in other studies, they are not critical to persistence within tomatoes.

**Table 2:** Competitive fitness of *Salmonella enterica* mutants in virulence, motility and attachment genes within red ripe tomatoes.

*S. enterica* genotype	Mean of the log competitive index ± standard error
*sirA3::cam* 1	0.244±0.044
*ΔcsrB20 ΔcsrC30::kan*	0.183±0.043
*flhDC::*Tn10	−0.133±0.094
*motA::* Tn10	0.338±0.101
*fliF:kan*	0.070±0.060
*hilA1550::*MudJ	0.334±0.172
14028 pSLT-	−0.038±0.122
***aroA::*** **Tn10** 2	**−1.251±0.183**
***hns::kan***	**N/A** 3
*ΔagfB36::frt ΔagfC37::kan*	−0.08±0.15
*ΔyihT27::kan*	0.05±0.07
*ΔyihT27-ompL19::frt*	−0.002±0.13

1 -underline indicates that the strain is more competitive than the wild type, based on a two tailed *t*-test (p<0.05) of log competitive indices as described in Materials and Methods.

2 -boldface indicates that the strain is less competitive than the wild type, based on a two tailed t-test (p<0.05) of log competitive indices as described in Materials and Methods.

3 - the *hns* mutant was completely unfit, none were recovered from tomatoes.

To test whether known *Salmonella* virulence genes have a role in colonization and persistence within tomato fruits, equal numbers of the wild type *Salmonella* 14028 and isogenic mutants were inoculated onto wounded tomato fruits (Table 2). Deletion of the entire virulence plasmid pSLT had no effect on the competitive fitness of *Salmonella* within red ripe tomatoes (Table 2). In addition to the *tra* and *spv* genes, pSLT carries *pef* and *rck* operons that are controlled by the *Salmonella N-*Acyl homoserine lactone receptor SdiA [39]. The lack of the phenotype for pSLT in these experiments is consistent with the earlier report that the *Salmonella* SdiA regulon is dispensable during persistence within tomato fruits [7].

In *Salmonella,* the global regulatory system BarA/SirA-Csr controls invasion genes located on *Salmonella* Pathogenicity Island (SPI)-1, SPI-4 and SPI-5 by activating transcription of small RNA genes *csrB* and *csrC* [40]–[42]. The BarA/SirA-Csr system, therefore, plays a role in some animal models of virulence and also is crucial for attachment to abiotic surfaces [40], [41], [43]. Furthermore, the *sirA* ortholog of *Erwinia amylovora* was upregulated during the infection of a pear fruit [15]. Our results indicated that the *sirA* mutant was 1.8-fold more competitive than the wild type in tomato (Table 2). Deletion of both regulatory *csr* sRNAs (which are known to be controlled by SirA [41], [43], [44]) resulted in a phenotype with a competitive index of 1.57, however this was not significantly different from either the wild type or a *sirA* mutation. In an LB broth co-culture with the wild type *S. enterica* sv Typhimurium 14028, competitive fitness of the *sirA* mutant was statistically indistinguishable from the fitness of a wild type strain marked with an antibiotic resistance gene in a neutral site of the genome (data not shown). Deletion of *hilA* tended to increase the competitive fitness of the mutant, consistent with the earlier reports for the function of SPI-1 effectors in endophytic fitness within alfalfa seedlings [38]. However, the *hilA* phenotype was highly variable between biological replicates and therefore was not statistically significantly different from the wild type overall (Table 2).

As an additional control, competitive fitness was also tested for the mutants in *hns,* a gene encoding a histone-like protein that is known to be expressed in tomatoes [7]) and *aroA,* a gene encoding 5-enolpyruvylshikimate 3-phosphate synthase. Both mutants were strongly outcompeted by the wild type in tomatoes, consistent with their pleiotropic phenotypes. However, in LB broth shake cultures under experimental conditions used in this study, competitive fitness of the mutants was not statistically different from the competitive fitness of the wild type marked with an antibiotic resistance (data not shown).

In IVET screens with plant pathogens, flagellar genes have not been detected as upregulated inside plant tissues [13]–[17]. Consistent with the reports that bacterial flagella elicit plant defense responses, flagellar genes reduced endophytic fitness of *S. enterica* sv. Typhimurium 14028 in alfalfa seedlings [38]. To test whether *Salmonella* flagella have a function in competitive persistence within tomato fruits, phenotypes of regulatory and structural flagellar mutants were compared. A mutation in *flhDC*, the master regulator of the flagellar regulon had no effect on competitive fitness within red ripe tomatoes (Table 2). Deletion of *fliF* results in a non-flagellated mutant with a functional motor, this mutant was as competitive as the wild type. Disruption of *motA* results in a flagellated non-motile mutant. The *motA* mutant colonized tomato fruit tissues better than the wild type (competitive index 2.57, Table 2) while in LB shake cultures, *motA* mutant was as competitive as the wild type (data not shown). The conclusion that the presence of a flagellum, even if it is not functional (as in the *motA* mutant) reduces competitive fitness of *Salmonella* in tomatoes is consistent with the reduced endophytic fitness of flagellated *Salmonella* strains within alfalfa sprouts [38].

## Discussion

Despite improvements in agricultural and management practices, leafy greens, tomatoes, cucurbits, peppers and nuts were among the foods linked to outbreaks of gastrointestinal illnesses caused by *Escherichia coli* O157:H7 and non-typhoidal serovars of *Salmonella enterica*, causing thousands of hospitalizations and multi-million dollar damage to the produce industry. This is consistent with an ability of enterics to colonize plants as alternate hosts [1]. However, it is not yet clear whether, how and to what extent enterics may have evolved to use plants as environmental reservoirs or alternate hosts.

If non-typhoidal salmonellae have indeed been selected to utilize plants as alternate hosts, then such evolution should result in effective colonization of plant tissues by these bacteria and in specifically altered patterns of bacterial gene expression in response to chemical signals and nutritional cues encountered during their colonization of plant tissues. The results presented here identify a significant set of genes in *S. enterica* sv. Typhimurium 14028 that are differentially regulated during colonization of the internal tissues of the tomato fruit. There appears to be no appreciable overlap of this set of “tomato-regulated” genes with the set of genes in *Salmonella* required for its infection and colonization of animals, or with the set of common genes in phytopathogenic bacteria required for their infection and colonization of plant tissues.

The differences in colonization of plant cultivars by different *Salmonella* serovars [28], [38], [45], [46] and the differences in responses of *Salmonella* RIVET reporters to the tomatoes of different cultivars (Fig. 4) raise the intriguing possibility that these interactions are co-evolved, with adaptations of each partner to the other. Previous studies have indicated that plant hosts recognize human enteric pathogens and initiate defenses to limit colonization by these bacteria. For example, PR1, a defense-related protein commonly used as a reporter of inducible plant defenses, was upregulated after inoculation with *Salmonella* in both *Arabidopsis* and in lettuce [6], [38]. Our results showed that the *cysB* reporter was differentially regulated in the tomato cultivars Hawaii 7997 and Bonny Best. The *cysB* gene contributes to the resistance of *Salmonella* swarms to antibiotics [33]. However, the connections between *cysB* expression and mechanisms of resistance in plants are not yet known. Further characterization of the genetic mechanisms that determine the specificity of interactions between *Salmonella* and crop cultivars may lead to the identification of plant genotypes that are less conducive to the proliferation of *Salmonella* within plant tissues and thus to enhanced food safety.

## Materials and Methods

### Strain construction

All strains are listed in Table S1. Deletion mutants were constructed using the one-step mutagenesis procedure of Datsenko and Wanner (2000) with primers listed in Tables S1 and S2. Mutants were confirmed by PCR and then transduced into the wild type *S. enterica* sv Typhimurium 14028 using phage P22-mediated transduction. When necessary, frt-kan-frt cassettes were removed as in [47], and confirmed by PCR with a set of outside primers (Table S2). The mutation in *cysB* was also confirmed phenotypically: the mutant was a cysteine auxotroph unable to utilize methionine, consistent with previous reports [48]. To construct TIM174, Δ*lpfA30,* Δ*fadH15*, ΔSTM2006 and Δ*cysB22::kan* mutations were introduced sequentially into JSG1748 using P22 phage transductions.

To construct RIVET reporters, putative promoters of interest were PCR amplified using primers specified in Table S2 using genomic DNA of *S. enterica* sv Typhimurium 14028 as a template. PCR products were first cloned into pCR2.1, and then sub-cloned into pGOA1193 [49]. The resulting constructs were sequenced at the University of Florida Biotechnology Core facilities, and then mated into *S. enterica* sv Typhimurium JS246. Integration was validated by PCR, using the “third” primer (see Tables S1, S2). As indicated in Table S1, some RIVET reporters were constructed using pCE70 or pCE71, which integrate into the frt scar generated by the excision of the frt-kan-frt cassette [29].

### Plant materials

All assays were initially conducted in unwaxed red ripe tomatoes cv. Campari purchased at local supermarkets. Follow-up experiments, as indicated in text, were carried out with green or red-ripe tomatoes of cvs. Bonny Best and Hawaii 7997 grown in a roof-top greenhouse. Plants were grown from seed in Miracle-Gro Potting Soil, fertilized biweekly with Miracle-Gro Tomato Plant Food (18-21–21) (Marysville, OH). Seeds of Hawaii 7997 were from Dr. Jeffrey B. Jones (Department of Plant Pathology, University of Florida). Seeds of cv. Bonny Best were purchased from Millington Seed Co (Millington, MI). Peppers and other tomato cultivars were grown in the field under organic-like conditions on a private farm outside of Archer, FL, from locally purchased seeds. Fruits were harvested individually from the field- and greenhouse-grown plants, ensuring that ripe and unripe fruit are from the same plants; they were inoculated with *Salmonella* within 3–4 hrs of harvest.

For experiments on *Salmonella* gene regulation in tomato phyllosphere, tomatoes cvs. Bonny Best and Hawaii 7997 were grown on a private farm outside of Archer, FL. Two days prior to the inoculation, secondary stems were excised from the field-grown plants, transferred to the laboratory, and were partially submerged into sterile de-ionized water in light at room temperature.

### Promoter-probe screen

**Figure 7:**
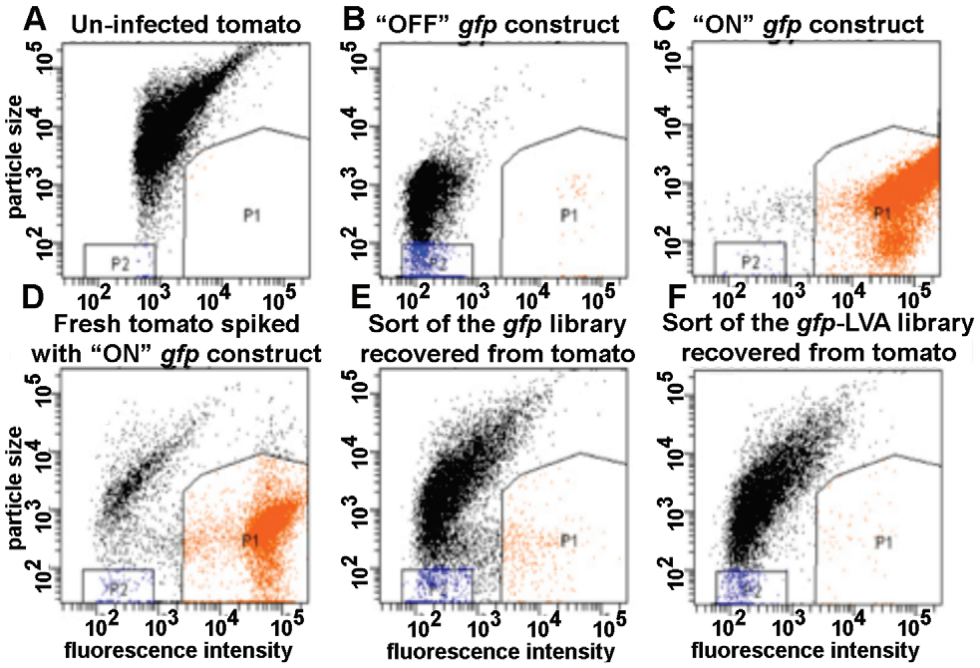
FACS cell sort of *Salmonella gfp* libraries recovered from tomato fruits. To establish parameters for cell sorting, filtered tomato pulp (A), “OFF” *gfp* construct (B) and the constitutive “ON” construct *dppA-gfp* (C) were injected into FACS-Aria. To confirm the selection of gates, P1 (“ON”) and P2 (“OFF”), a *dppA-gfp* reporter was mixed with tomato pulp and injected into FACS-Aria (D). Once gates were established, promoter probe libraries recovered from tomatoes were sorted (E, F). Cells segregating into gates P1 (“ON”) or P2 (“OFF”) were collected for the identification of promoters.

To harvest promoters that were differentially regulated inside red ripe Campari tomatoes, two promoter probe-*gfp* libraries were used [50]. One library was constructed with stable Turbo-GFP (Evrogen, Inc) and another library was constructed with Turbo-GFP that was destabilized by an LVA C-terminal tag [50]. Libraries were transformed back into *S. enterica* sv Typhimurium 14028 by electroporation. Fluorescent activated cell sorting (FACS) was done on FACS Aria-I cell sorter (BD Biosciences, San Jose, CA) supported by Diva 6.2 software. Green fluorescent cells were detected at 488 nm with 525/10 band path filter. Conditions for the cell sort were established first by defining the parameters for pTURBO-*gfp* (non-fluorescent negative control), blended filtered tomato pulp (negative control), a strongly fluorescent construct carrying promoter for *dppA* (positive control) and filtered tomato pulp spiked with the *dppA-gfp* construct (Fig. 7).

For the fluorescence-activated cell sorts, libraries were grown in LB broth or in M9 medium with ampicillin and glucose as the sole carbon source. The libraries were then sorted to remove constructs that were strongly expressed *in vitro*. “Dim” constructs were collected, washed in PBS to remove the carrier buffer and then inoculated into shallow wounds made in red ripe tomatoes cv. Campari or field grown tomatoes as described in [7]. Infected tomatoes were incubated for 2 days at room temperature; fruits were then ground on ice in Phosphate-Buffered Saline (PBS) with a mortar and pestle. Pulp was filtered through Whatman #1 filter paper. The resulting suspension was then centrifuged at 24,000 g for 10 min, the supernatant was removed and the pellet was resuspended in 7 ml of PBS. The resulting suspension was then sorted into the P1 gate (Fig. 7) to isolate fluorescent constructs. These were plated on LB with ampicillin. Six hundred individual colonies resulting from three independent experiments were picked and transferred into wells of microtiter 96-well plates. Lack of strong fluorescence during growth in LB broth or in M9 with glucose was confirmed with a multimode Victor-2 microtiter plate reader. Colony PCR with primers Insert1R and Turbo4F was carried out to identify unique constructs. Plasmids extracted from 288 individual colonies were individually transformed into chemically competent *E. coli* DH5a, from which they were extracted using Qiagen Plasmid miniprep kit and then submitted for sequencing with either Insert1R or Turbo4F.

### Culture conditions and reporter assays

Strain construction is described above, strains are listed in Table S1, additional constructs [51]–[53] were shared by colleagues. All strains were maintained as frozen glycerol stocks, and were sub-cultured into LB with appropriate antibiotics (50 µg/ml kanamycin, 200 µg/ml ampicillin, and 10 µg/ml tetracycline) prior to the experiments. For plate assays, bacteria were seeded into soft LB or M9 agar (0.3% agar) with or without X-gal (40 µg/ml) as indicated in text. Linoleic acid purchased from MP Biomedical, Solon, OH, USA.

For the RIVET assays in tomatoes, *Salmonella* cultures were grown at 37°C overnight in LB supplemented with tetracycline. Bacterial cultures were then pelleted, washed three times in an equal volume of sterile PBS and diluted to 10^8^ cfu/ml. Approximately 10^5^ cfu (in 3 µl of PBS) were inoculated onto superficial 1 mm wounds on surfaces of unwaxed immature or mature fruits. At least three technical and six biological replications were carried out for each experiment. All RIVET assays were carried out for a week. To harvest samples, unless otherwise indicated in text, cores 15 mm×0.5 mm were removed from fruits, homogenized in PBS and aliquots were then plated on xylose-lysine deoxycholate (XLD) agar (Oxoid). Individual colonies were patched on LB agar with tetracycline to detect constructs in which TnpR recombinase was active. Regulation of the reporters in tomato phyllosphere was carried essentially as above, except 10 µl of the bacterial suspension in sterile water were spotted onto the abaxial surface of tomato leaves and incubated in light at room temperature for two days. Leaves were then gently blotted on XLD, and then assayed for the TnpR recombinase activity.

### Fitness of the *Salmonella* mutants

To calculate a competitive index, wild type *S. enterica* sv. Typhimurium 14028 and isogenic mutants were seeded at 10^5^ cfu/infection, roughly at a1:1 ratio into tomatoes. In parallel, *S. enterica* sv. Typhimurium 14028 and its isogenic tetracycline-resistant derivative JS246 were similarly inoculated onto eight tomato fruits, two wounds per fruit. To establish *in vitro* baselines, individual mutants and the wild type (strain ATCC 14028) were grown in LB to OD_600_ ∼ 0.3–0.9, washed in PBS, diluted to OD_600_ ∼ 0.05 in LB, mixed 1∶1 and incubated for 3 hrs at 22°C in 5 ml tubes on a shaker (160 rpm). All samples were incubated and harvested using similar protocols. The relative ratios of the strains in the inocula and in the recovered samples were calculated by dilution plating and patching on antibiotic containing media. Competitive indices were calculated for each treatment using the formula (M_out_/WT_out_)/(M_in_/WT_in_), where M is the proportion of mutant cell and WT is the proportion of the wild type cells in the inocula (_in_) or in the recovered samples (_out_). Statistical and biological significance of each competitive index were established by comparing log values of the competitive indices of each pair to the log of competitive index similarly calculated for ATCC 14028 vs JS246 (0.03±0.089), using a two-tailed *t*-test with unequal variances (p<0.05).

### Extraction of hydrophobic components from red tomatoes

Tomatoes cv. Campari were blended and mixed with two volumes of chloroform. The organic phase was washed with 50∶50 ethanol:water, rotary evaporated to dryness and re-dissolved in chloroform. For bioassays, aliquots were dried down in a Spin/Vac and then resuspended in canola oil. Approximately 0.4 tomato equivalents were added to the LB 0.3% agar plates for the bioassays.

## Supporting Information

Table S1 Strains and Plasmids used in the study.
(0.11 MB DOC)

Table S2 Primers used in the study.
(0.07 MB DOC)
