# Supplementary material for: Specific Responses of Salmonella enterica to Tomato Varieties and Fruit Ripeness Identified by In Vivo Expression Technology
Source: PLoS One. 2010 Aug 31;5(8):e12406. doi: 10.1371/journal.pone.0012406 (PMC2930847; doi:10.1371/journal.pone.0012406)
Supplement: Table S1 — Strains and Plasmids used in the study. (0.11 MB DOC) [file pone.0012406.s001.doc]

**Table S1. Strains and plasmids used in this study.**

| **Strain Name** | **Genotype or description** | **Source, construction or reference** |
| --- | --- | --- |
| ***E. coli*** |  |  |
| BW20767 | *leu-63* :: IS*10 recA1 creC510 hsdR17 endA1 zbf-5 uidA*(MluI) :: *pir*^+^ *thi* RP4-2-tet :: ∆Mu-1kan :: Tn*7* | [51] |
| DH5α | F– ∆ (*lacZYA-argF)U169 recA1 endA1 hsdR17 supE44 thi-1 gyrA96 relA1* | Life Technologies |
| DH5α λpir | F– ∆ (*lacZYA-argF)U169 recA1 endA1 hsdR17 supE44 thi-1 gyrA96 relA1* λ:: *pir* | [52] |
|  |  |  |
| ***Salmonella enterica serovar* Typhimurium** | |  |
| 14028 | wild type | American Type Culture Collection |
| AT343 | 14028 *motA*::Tn10 | R. Harshey, unpublished data |
| AT351 | 14028 *flhD*::Tn10 | [44] |
| BA1550 | 14028 *hilA1550*::MudJ | [40] |
| BA746 | 14028 *sirA3*::cam | [40] |
| BA770 | 14028 pSLT- | [39] |
| JS246 | 14028 *yjeP*::*res1-tetRA-res1* | [29] |
| JSG1748 | 14028 ∆ *bcs*::kan | [37] |
| JTN24 | JS246 *fadH^+^ fadH*'::*tnpR-lacZY* | JS246 mated with BW20767 pGOA-fadH'. Integration of the plasmid confirmed by PCR with primers JTN3 and MT59. |
| JTN26 | JS246 *dgt*^+^ *dgt*'::*tnpR-lacZY* | [7] |
| JTN69 | JS246 STM2006^+^ STM2006'::*tnpR-lacZY* | JS246 mated with BW20767 pGOA-STM2006. Integration of the plasmid confirmed by PCR with primers JTN15 and MT59. |
| JTN71 | JS246 *cysB^+^ cysB*::*tnpR-lacZY* | JS246 mated with BW20767 pGOA-cysB. Integration of the plasmid confirmed by PCR with primers JTN26 and MT59. |
| JTN97 | 14028 ∆ *fadH15*::kan | Constructed by Datsenko and Wanner mutagenesis using PCR product amplified with JTN60 and JTN61 |
| JTN112 | JS246 STM2608*^+^* STM2608'::*tnpR-lacZY* | JS246 mated with BW20767 pGOA-STM2608. Integration of the plasmid confirmed by PCR with primers JTN41 and MT59. |
| JTN113 | JS246 *lpfA^+^* as*-lpfA*::*tnpR-lacZY* | JS246 mated with BW20767 pGOA-as-lpfA*.*  Integration of the plasmid confirmed by PCR with primers JTN50 and MT59. |
| JTN114 | JS246 STM4429*^+^* STM4429'::*tnpR-lacZY* | JS246 mated with BW20767 pGOA-STM4429. Integration of the plasmid confirmed by PCR with primers JTN44 and MT59. |
| JTN129 | JS246 *fliQ^+^* *fliQ'*::*tnpR-lacZY* | JS246 mated with BW20767 pGOA-*fliQ.* Integration of the plasmid confirmed by PCR with JTN57 and MT59. |
| JTN131 | JS246 *ilvA^+^* *ilvA'*::*tnpR-lacZY* | JS246 mated with BW20767 pGOA-ilvA. Integration of the plasmid confirmed by PCR with primers JTN47 and MT59. |
| JTN153 | 14028 ∆ *cysB22*::kan | Constructed by Datsenko and Wanner mutagenesis, using PCR product amplified with JTN70 and JTN71 |
| JTN169 | 14028 ∆ *fadH15*::kan | 14028 X P22/JTN97 |
| JTN170 | 14028 ∆ *cysB22*::kan | 14028 X P22/JTN153 |
| JTN175 | 14028 ∆ *lpfA30*::kan | 14028 X P22/MM_008-C03 |
| JTN178 | 14028 ∆ STM2006::kan | Constructed by Datsenko and Wanner mutagenesis, using PCR product amplified with JTN72 and JTN73 |
| JTN180 | 14028 ∆ STM2006::kan | 14028 X P22/JTN178 |
| JTN186 | JS246 *jtnB*^+^ *jtnB*'::*tnpR-lacZY* | JS246 mated with BW20767 pGOA-jtnB. Integration of the plasmid confirmed by PCR with primers JTN111 and MT59 |
| JTN187 | JS246 *sraF25*::kan | Constructed by Datsenko and Wanner mutagenesis, using PCR product amplified with JTN127 and JTN128 |
| JTN188 | JS246 *sraF25*::kan | JS246 P22/JTN187 |
| JTN196 | JS246 *sraF25*::frt | Made by electroporating pCP20 into JTN188 |
| JTN197 | JS246 *sraF25*::*tnpR-lacZY* | Made by electroporating pCE71 into JTN196 |
| JTN198 | JS246 *hilA29*::kan | Constructed by Datsenko and Wanner mutagenesis, using PCR product amplified with JTN140 and JTN141 |
| JTN199 | JS246 *hilA29*::frt | Made by electroporating pCP20 into JTN198 |
| JTN200 | 14028 ∆ *yihT27-tnpR-lacZ* | Made by electroporating pCPE70 into TIM2267 pCP20 |
| JTN201 | JS246 *hilA29-tnpR-lacZ* | Made by electroporating pCE70 into JTN199 pCP20 |
| JTN202 | 14028 ∆*agfC37-tnpR-lacZ* | Made by electroporating pCE70 into TIM2264 pCP20 |
| JTN203 | 14028 ∆*yihT27-tnpR-lacZ yjeP*::*res1-tetRA-res1* | JTN200 X P22/JS246 |
| JTN204 | 14028 ∆*agfC37-tnpR-lacZ yjeP*::*res1-tetRA-res1* | JTN202 X P22/JS246 |
| KK649 | 14028 ∆ *fliF*::kan | K. Klose, unpublished data |
| MM_003-E04 | LT2 ∆*agfB36*::kan | [53] |
| MM_006-D05 | LT2 ∆*agfA35*::kan | [53] |
| MM_006-E05 | LT2 ∆*agfC37*::kan | [53] |
| MM_008-C03 | LT2 ∆*lpfA30*::kan | [53] |
| MM_015-E06 | LT2 ∆*ompL19*::kan | [53] |
| MM_015-F06 | LT2 ∆*yihT27*::kan | [53] |
| TIM111 | 14028 ∆*csrB20* ∆*csrC30*::kan | [46] |
| TIM174 | 14028 ∆*bcs* ∆ *lpfA30* ∆ *fadH15* ∆ STM2006 ∆*cysB22*::kan | See Materials and Methods |
| TIM2260 | 14028 ∆*agfB36*::kan | 14028 X P22/MM_003-E04 |
| TIM2261 | 14028 ∆*agfC37*::kan | 14028 X P22/MM_006-E05 |
| TIM2262 | 14028 ∆*ompL19*::kan | 14028 X P22/MM_015-E06 |
| TIM2263 | 14028 ∆*yihT27*::kan | 14028 X P22/MM_015-F06 |
| TIM2264 | 14028 ∆*agfC37*::frt | Made by electroporating pCP20 into TIM2261 |
| TIM2265 | 14028 ∆*agfB36*::frt | Made by electroporating pCP20 into TIM2260 |
| TIM2266 | 14028 ∆*ompL19*::frt | Made by electroporating pCP20 into TIM2262 |
| TIM2267 | 14028 ∆*yihT27*::frt | Made by electroporating pCP20 into TIM2263 |
| TIM2268 | 14028 ∆*agfB36*::frt ∆*agfA35*::kan | TIM2265 X P22/TIM2264 |
| TIM2270 | 14028 ∆*ompL19*::frt ∆*yihT27*::kan | TIM2266 X P22/TIM2267 |
| TIM2272 | 14028 ∆*ompL19-yihtT27*::frt | Made by electroporating pCP20 into TIM2270 and selecting for desired product with PCR. Deletion confirmed by sequencing. |
|  |  |  |
| ***Salmonella enterica serovar* Montevideo LJH519** | Human isolate from a *Salmonella* outbreak in tomatoes. | Linda Harris |
| ***Salmonella enterica serovar* Javiana ATCC BAA-1593** | Human stool, collected during *Salmonella* outbreak in Pennsylvania linked to fresh Roma tomatoes. | American Type Culture Collection |
| ***Salmonella enterica serovar* Newport C6.3** | Isolated from a tomato field on the Eastern Shore of Virginia | Keith Schneider |
| ***Salmonella enterica serovar* Braenderup 04E01347** | Human stool, isolated from a Roma tomato outbreak. | Pennsylvania Department of Health |
| ***Salmonella enterica serovar* Braenderup 04E01556** | Human stool, isolated from a Roma tomato outbreak. | Pennsylvania Department of Health |
| ***Salmonella enterica serovar* Braenderup 04E00783** | Human stool, isolated from a Roma tomato outbreak. | Pennsylvania Department of Health |
|  |  |  |
| **Plasmid Name** | **Description** | **Construction or source** |
| pCP20 | FLP^+^, λ cI857^+^, λ p_R_ Rep^ts^, amp, cm | [29] |
| pGOA-as-lpfA | as-*lpfA-tnpR* fusion in pGOA1193 | Contains PCR fragment amplified with JTN48 and JTN49 |
| pGOA-cysB' | *cysB'-tnpR* fusion in pGOA1193 | Contains PCR fragment amplified with JTN24 and JTN25 |
| pGOA-fadH' | *fadH'-tnpR* fusion in pGOA1193 | Contains PCR fragment amplified with JTN1 and JTN2 |
| pGOA-fliQ' | *fliQ'-tnpR* fusion in pGOA1193 | Contains PCR fragment amplified with JTN57 and JTN58 |
| pGOA-ilvA' | *ilvA'-tnpR* fusion in pGOA1193 | Contains PCR fragment amplified with JTN45 and JTN46 |
| pGOA-STM2006' | STM2006'-*tnpR* fusion in pGOA1193 | Contains PCR fragment amplified with JTN13 and JTN14 |
| pGOA-STM2608' | STM2608'*-tnpR* fusion in pGOA1193 | Contains PCR fragment amplified with JTN39 and JTN40 |
| pGOA-STM4429' | STM4429'-*tnpR* fusion in pGOA1193 | Contains PCR fragment amplified with JTN42 and JTN43 |
| pGOA1193 | pIVET5n *tnpR (*amp) | [49] |
| pKD4 | FRT-kan-FRT template (amp oriR6K) | [47] |
| pKD46 | Lambda Red^+^ (amp pSC101 oriTS) | [47] |
| pCE70 | FRT-*tnpR*-*lacZY* this oriR6K (kan). Contains wild type *tnpR* Shine Dalgarno. FRT orientation A. | [29] |
| pCE71 | FRT-*tnpR-lacZY* this oriR6K (kan). Contains wild type *tnpR* Shine Dalgarno. FRT orientation B. | [29] |
