# Supplementary material for: Specific Responses of Salmonella enterica to Tomato Varieties and Fruit Ripeness Identified by In Vivo Expression Technology
Source: PLoS One. 2010 Aug 31;5(8):e12406. doi: 10.1371/journal.pone.0012406 (PMC2930847; doi:10.1371/journal.pone.0012406)
Supplement: Table S2 — Primers used in the study. (0.07 MB DOC) [file pone.0012406.s002.doc]

**Table S2**. Primers used in this study.

| Name | DNA sequence | NC_003197 Coordinates | Additional information |
| --- | --- | --- | --- |
| Insert1R | CCACCAGCTCGAACTCCAC | N/A | Binds within Turbo-PRL1 and Turbo-PRL3 [50]. |
| JTN1 | CTCGAGAATTTGTCGACAACGAAACGGGCT | 3385885 :3385908 | Xho1 cutting sites indicated by underline |
| JTN109 | CTCGAGAAAGGTCGTAAGGATACTCAGCGA | 3565885 :3565908 | Xho1 cutting sites indicated by underline |
| JTN110 | CTCGAGTGTACAAACGGCTTTTACTTACGC | 3566243 :3566266 | Xho1 cutting sites indicated by underline |
| JTN111 | CTGGGCCTTTTTGCATTTATCTCT | 3566404 :3566427 |  |
| JTN13 | CTCGAGAAGAGTATCTCGATGGTACCTGCG | 2084879 :2084902 | Xho1 cutting sites indicated by underline |
| JTN14 | CTCGAGTATGGACGTTCAAAGCCCGTAAGT | 2085509 :2085532 | Xho1 cutting sites indicated by underline |
| JTN15 | CTATCGACACGTTCGGTACCAAAG | 2084849 :2084872 |  |
| JTN2 | CTCGAGACCAGGTGCGGCTGATAGCTGTAA | 3386575 :3386598 | Xho1 cutting sites indicated by underline |
| JTN24 | CTCGAGTTTATCTGCAGACTTAATCGGGCT | 1809417 :1809440 | Xho1 cutting sites indicated by underline |
| JTN25 | CTCGAGAAGTGGGTTGAAGGCAAGAAATAA | 1809792 :1809815 | Xho1 cutting sites indicated by underline |
| JTN26 | AACGAAGCTGCTGCAATTTCATAA | 1809360 :1809383 |  |
| JTN3 | TCCTGGATGGTTTTCGACAACTTG | 3385801 :3385824 |  |
| JTN39 | CTCGAGGCGACATATTTTTTCAGATCCCCT | 2755460 :2755483 | Xho1 cutting sites indicated by underline |
| JTN40 | CTCGAGTTCGGTCTGAAATGGGAGAAAAGC | 2756723 :2756746 | Xho1 cutting sites indicated by underline |
| JTN41 | ATTATTACGGACCAGATCATCAGC | 2755211 :2755234 |  |
| JTN42 | CTCGAGATTTTTGTTTCACCGTTGCGATGA | 4672464 :4672487 | Xho1 cutting sites indicated by underline |
| JTN43 | CTCGAGTTGAGGCCGGTTTACAGAATAATA | 4673103 :4673126 | Xho1 cutting sites indicated by underline |
| JTN44 | TCAACAGATCGCTCAGATGCAACT | 4672212 :4672235 |  |
| JTN45 | CTCGAGGGCGATGAAAGATCTGTTTGAAGA | 4114655 :4114678 | Xho1 cutting sites indicated by underline |
| JTN46 | CTCGAGTGGATATCAGGCCCACTTTACTTT | 4115411 :4115434 | Xho1 cutting sites indicated by underline |
| JTN47 | TCTGGAAAACGTCTCGCAAAATGA | 4115492 :4115515 |  |
| JTN48 | CTCGAGCCAGTGCTGTGCAAGATATCAAAC | 3827328 :3827351 | Xho1 cutting sites indicated by underline |
| JTN49 | CTCGAGAGGTTGTTTTTGCTCTGTCTGCTC | 3827943 :3827966 | Xho1 cutting sites indicated by underline |
| JTN50 | AAATAACACGCGTGCTGCTTAATG | 3827262 :3827285 |  |
| JTN57 | CTCGAGCATCCCTTTTTTGATTATCGACCT | 2065895 :2065918 | Xho1 cutting sites indicated by underline |
| JTN58 | CTCGAGCTGAACAGAGTGCGCACGTAATCC | 2066294 :2066317 | Xho1 cutting sites indicated by underline |
| JTN59 | AATAGAAAAAAGCGGCGTATCGTT | 2066531 :2066554 |  |
| JTN60 | GCTACAGTAGTAACTCATCCGACCACATAACAATAATTTTACATACT**TGTAGGCTGGAGCTGCTTCG** | 3386198 :3386244 | Priming sites for pKD4 indicated in bold |
| JTN61 | TATGACGGCGTAAGCCCGATAAGCGTAGCGCCATCAGGCAAAGTCG**CATATGAATATCCTCCTTAG** | 3388274 :3388319 | Priming sites for pKD4 indicated in bold |
| JTN70 | AAAGGCGGCTTTGTCTGCCATGCCACTACGACACAAACCGACGGTGATA**TGTAGGCTGGAGCTGCTTCG** | 1808358 :1808406 | Priming sites for pKD4 indicated in bold |
| JTN71 | TGTCGCTCACCCCAGTCACATCGTAATCTATGCTTCTGGGAACTCACTC**CATATGAATATCCTCCTTAG** | 1809603:1909651 | Priming sites for pKD4 indicated in bold |
| JTN72 | AGCCAAAGAGCATACGCCGGAGACTGTTCGTGTTCTCAAAATCACAGGA**TGTAGGCTGGAGCTGCTTCG** | 2085350 :2085398 | Priming sites for pKD4 indicated in bold |
| JTN73 | AAAGACAGATATTGCGTCCCTGAAGTCTCGCGTACTGCTGAAGTAGCGA**CATATGAATATCCTCCTTAG** | 2086133:2086181 | Priming sites for pKD4 indicated in bold |
| JTN127 | TAACTTCATTGCCGGTCGATCGTCATTACGATGTGTGCAAA**TGTAGGCTGGAGCTGCTTCG** | 3392080 :3392120 | Priming sites for pKD4 indicated in bold |
| JTN128 | CTGACGCATTCCTTTATGGCTGCCCGGTGACCGGATGTGGT**CATATGAATATCCTCCTTAG** | 3392121 :3392161 | Priming sites for pKD4 indicated in bold |
| JTN140 | TGGTTCAAAAGATGGAAACAGGATCCCCGCTTGATTAAATTACGGTAA**TGTAGGCTGGAGCTGCTTCG** | 3021470 :3021517 | Priming sites for pKD4 indicated in bold |
| JTN141 | ACCAGATTACGATGATAAAAAAATAATGCATATCTCCTCTCTCAGATT**CATATGAATATCCTCCTTAG** | 3021518 :3021565 | Priming sites for pKD4 indicated in bold |
| MT59 | CAAAAAGTCGCATAAAAATTTATCC | N/A | Binds within *tnpR* of pGOA1193 [7]. |
| Turbo4F | AAAGTGCCACCTGACGTCT | N/A | Binds within Turbo-PRL1 and Turbo-PRL3 [50]. |
